# Platelet signaling at the nexus of innate immunity and rheumatoid arthritis

**DOI:** 10.3389/fimmu.2022.977828

**Published:** 2022-11-25

**Authors:** Steven Z. Jiang, Jeffrey L. To, Michael R. Hughes, Kelly M. McNagny, Hugh Kim

**Affiliations:** ^1^ Centre for Blood Research, University of British Columbia, Vancouver, BC, Canada; ^2^ Department of Biochemistry and Molecular Biology, University of British Columbia, Vancouver, BC, Canada; ^3^ School of Biomedical Engineering, University of British Columbia, Vancouver, BC, Canada; ^4^ Department of Medical Genetics, University of British Columbia, Vancouver, BC, Canada; ^5^ Department of Oral Biological and Medical Sciences, University of British Columbia, Vancouver, BC, Canada

**Keywords:** platelets, rheumatoid arthritis, inflammation, cell signaling, cytokines

## Abstract

Rheumatoid arthritis (RA) is a debilitating autoimmune disorder characterized by chronic inflammation of the synovial tissues and progressive destruction of bone and cartilage. The inflammatory response and subsequent tissue degradation are orchestrated by complex signaling networks between immune cells and their products in the blood, vascular endothelia and the connective tissue cells residing in the joints. Platelets are recognized as immune-competent cells with an important role in chronic inflammatory diseases such as RA. Here we review the specific aspects of platelet function relevant to arthritic disease, including current knowledge of the molecular crosstalk between platelets and other innate immune cells that modulate RA pathogenesis.

## Introduction: RA pathophysiology

Rheumatoid arthritis (RA) is an autoimmune disease that affects ~0.4% of the global population (1). The disease is characterized by chronic inflammation of the joints, degradation of bone and cartilage (2), and progressive pain and loss of mobility such that 37% of RA patients become disabled (3). RA decreases life expectancy and is associated with increased risk of lymphoma, cardiovascular disease, and lung cancer (4, 5). The precise etiology of RA is unknown, but genetic predisposition (6), abnormal DNA methylation patterns (7), smoking (8), infection (9), and gut microbiome dysbiosis (10) contribute to RA development. A classic hallmark of RA is the presence of the self-antigen binding anti-citrullinated protein antibodies (ACPA) that trigger chronic activation of innate immune cells. Citrullination is a protein post-translational modification where the amino acid arginine is substituted for citrulline, leading to the generation of ACPAs that initiate a pro-inflammatory response (11).

During the onset of RA, the innate immune response recruits platelets and platelet microparticles (PMPs) (12, 13) as well as leukocytes (14) to the joints. PMPs and leukocytes are a source of pro-inflammatory chemokines and cytokines (15) that signal to the resident fibroblast-like synoviocytes (FLS) lining the synovial cavity (16). FLS hyper-proliferate to form a tumor-like structure called a pannus (2, 17). Angiogenesis lays down new blood vessels that supply nutrients (18) and allow the pannus to grow until it reaches the bone and cartilage where “activated” and increasingly aggressive FLS secrete joint-destroying matrix metalloproteinases (MMP) including MMP-1, MMP-3 and MMP-13 (2, 17). Importantly, the distinct roles of platelets in mediating the innate immune response are now increasingly recognized in the context of RA.

## Platelets are innate immune cells that drive RA pathogenesis

**Figure 1:**
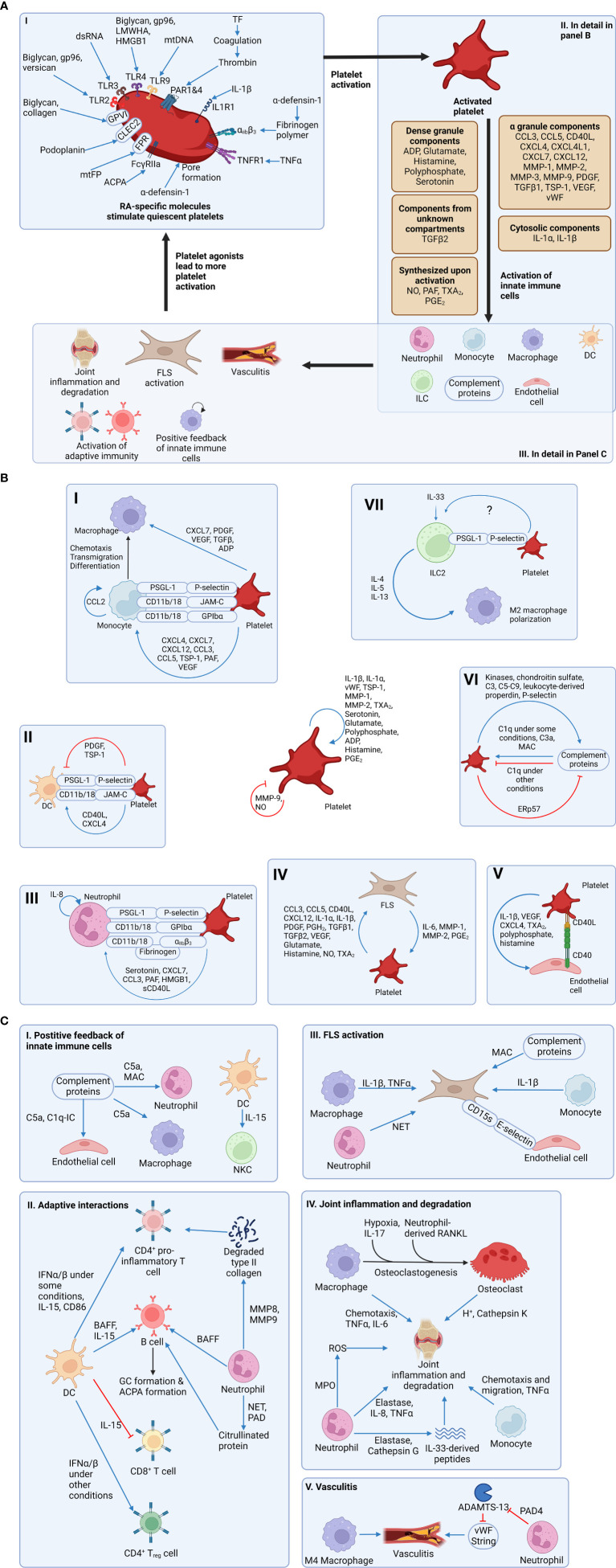
Platelet signaling contributes to the activation of innate immunity and the development of rheumatoid arthritis (RA). **(A)** Section I: RA-specific molecules activate platelets by binding to receptors on the surface of platelets. Notably, α-defensin mediates its action by forming a pore on the plasma membrane of platelets. Section II: Activated platelets secrete mediators stored in dense granules and α-granules. Notably, some mediators are freshly synthesized upon platelet activation and therefore not stored in granules. Also, IL-1 is stored in the cytosol of platelets, while TGF-β is stored in an unknown subcellular compartment. Regardless of the subcellular localization, these mediators contribute to the activation of more platelets and other cell types. Section III: Activated innate immune cells lead to more immune activation through a positive feedback loop and the generation of RA symptoms. Also, FLSs, which are activated directly and indirectly by platelets, can activate more platelets, resulting in a vicious cycle. **(B)** Platelets can activate many other cell types. First, platelets are capable of autocrine-activation and feedback inhibition. Section I: Platelets can support monocyte transmigration using adhesion molecules. Also, platelet-derived soluble mediators can activate monocytes. After monocytes transmigrate into subendothelial space and differentiate into macrophages, platelets can activate macrophages. Section II: Platelets support DC transmigration into the joint using adhesion molecules. Some platelet-derived soluble mediators activate DCs, while other mediators inhibit DCs. Section III: Platelets activate neutrophils using soluble mediators and support their transmigration using adhesion molecules. Section IV: Platelets and FLSs can activate each other using soluble mediators, resulting in a positive feedback loop. Section V: Activated platelets can activate endothelial cells. Section VI: Activated platelets and complement proteins can activate each other, but feedback inhibition exists in both directions. Section VII: ILC2 express PSGL-1 and adhere to platelets (likely through P-selectin) in select tissues. Platelet interaction with ILC2s potentiates their response to IL-33 and survival and/or proliferation, but it is not known if this interaction occurs in synovial tissue. Also, the mechanism of platelet-mediated enhanced ILC2 function has not been determined. ILC2s ameliorate RA pathogenesis by limiting pro-inflammatory M1 polarization and promoting M2 polarization. **(C)** Activated innate immune cells result in more immune activation and the development of RA symptoms. Section I: Complement proteins and DCs can activate other innate immune cells. Section II: Both neutrophils and DCs can activate proinflammatory subsets of CD4^+^ T cell and B cells. Notably, neutrophils activate B cells and T cells not only by releasing soluble mediators, but also by generating citrullinated proteins and degraded type II collagen, both of which serve as autoantigens. Activated proinflammatory CD4^+^ T cells and B cells can promote RA progression. However, DCs inhibit CD8^+^ T cell cytotoxicity and induce CD4^+^ T_reg_ differentation, thereby inhibiting RA progression. Therefore, the role of DCs in RA is complex. Section III: Macrophages, monocytes, complement proteins, and neutrophils activate a pro-inflammatory and pro-joint degrading phenotype of FLS. Endothelial cell-derived E-selectin allows these pro-inflammatory FLS to invade distant joints. Section IV: Macrophages can differentiate into osteoclasts in RA joints. Macrophages, osteoclasts, neutrophils, and monocytes contribute to joint inflammation and degradation. Notably, neutrophil-derived elastase and cathepsin G can cleave IL-33 into three proinflammatory peptides, which are more potent. Section V: M4 macrophages contribute to vasculitis in RA. ADAMTS-13 normally cleaves vasculitis-promoting vWF strings. Neutrophil PAD4 inhibits ADAMTS-13, thereby promoting vasculitis. Figures were made using BioRender.com. α_iib_β_3_, integrin alpha iib beta 3; ACPA, anti-citrullinated protein antibody; ADAMTS-13, a disintegrin and metalloproteinase with a thrombospondin type 1 motif member 13; ADP, adenosine diphosphate; BAFF, B cell activating factor; CCL, chemokine C-C motif ligand; CD, cluster of differentiation; CLEC2, C-type lectin-like receptor 2; CXCL, chemokine C-X-C motif ligand; CXCL4L1, non-allelic variant of CXCL4; C1q, complement component 1q; C1q-IC, a complex of C1q and immune complex (which is an antibody-antigen complex); C3, complement components 3; C5a, complement component 5a; C5-C9, complement components 5, 6, 7, 8, and 9; DC, dendritic cell; ERp57, endoplasmic reticulum protein 57; FcγRIIa, Fc-gamma receptor IIa; FLS, fibroblast-like synoviocyte (also known as synovial fibroblast); FPR, formyl peptide receptor; GP, glycoprotein; gp96, heat shock protein 96; HMGB1, high mobility group box protein 1; IFN, interferon; IL, interleukin; ILC, innate lymphoid cell; JAM-C, junctional adhesion molecule-C; LMWHA, depolymerized low molecular weight hyaluronic acid; MAC, membrane attack complex; MMP, matrix metalloproteinase; MPO, myeloperoxidase; mtDNA, extracellular mitochondrial DNA; mtFP, extracellular mitochondrial formyl peptide; M4 macrophage, a subset of macrophage induced by CXCL4; NET, neutrophil extracellular trap; NKC, natural killer cell; NO, nitric oxide; PAD, protein arginine deiminase; PAF, platelet activating factor; PAR1&4, protease activated receptor 1 and protease activated receptor 4; PDGF, platelet-derived growth factor; PGE_2_, prostaglandin E_2_; PGH_2_, prostaglandin H_2_; PSGL-1, P-selectin glycoprotein ligand 1; RA, rheumatoid arthritis; RANKL, receptor activator of nuclear factor κB ligand; ROS, reactive oxygen species; sCD40L, soluble CD40 ligand; TGF, transforming growth factor; Th1, T helper 1 cell; Th17, T helper 17 cell; TNF, tumor necrosis factor; TNFR1, tumor necrosis factor receptor 1; TSP-1, thrombospondin 1; TXA_2_, thromboxane A_2_; VEGF, vascular endothelial growth factor; vWF, von Willebrand factor.

Platelets are small (2-3 μm) anucleate blood cells responsible for hemostasis (19). Resting platelets are activated by many ligands, such as collagen and thrombin, *via* platelet surface receptors (**Figure 1A**). Activated platelets change shape, aggregate and secrete granule contents that include prothrombotic mediators, cytokines, chemokines and growth factors (20), resulting in thrombus formation at the site of injury (**Table 1**). The process leading to activation is typically tightly regulated, allowing most platelets to circulate in their quiescent states under normal physiologic conditions. However, there is an increasing body of evidence suggesting that dysregulation of platelet activation may be a key factor in driving various chronic inflammatory diseases, including RA (101).

**Table 1:** Platelet-derived mediators with documented role(s) in RA pathogenesis.

Mediator	Target cells	Role(s) in RA pathogenesis	Refs
**CCL3**	B cell, monocyte, macrophage, neutrophil, FLS	Immune cell recruitment and activation, autoantibody production, pro-inflammatory cytokine production	(21–25)
**CCL5**	Monocyte, B cell, CD4^+^ T cell, FLS	Immune cell recruitment and activation, pro-inflammatory cytokine production, autoantibody production	(23, 26–30)
**CD40L &** **sCD40L**	Neutrophil, DC, endothelial cell, B cell, FLS	Endothelium activation and leukocyte recruitment, DC maturation, angiogenesis, pro-inflammatory cytokine production, ROS production, autoantibody production	(31–36)
**CXCL4**	Monocyte, macrophage, DC, neutrophil, endothelial cell, CD4^+^ and CD8^+^ T cell	Immune cell recruitment and activation, endothelium activation, ROS and pro-inflammatory cytokine production	(37–47)
**CXCL7**	monocyte, neutrophil, endothelial cell	Immune cell recruitment and activation, ROS production	(37, 46, 48, 49)
**CXCL12**	Pre-B cell, CD4^+^ and CD8^+^ T cell, Monocyte, FLS	Immune cell recruitment and activation, proinflammatory cytokine production	(30, 50–53)
**IL-1α &** **IL-1β**	FLS, endothelial cell	Endothelial activation and immune cell recruitment, pro-inflammatory cytokine secretion, osteoclastogenesis, angiogenesis, FLS proliferation	(12, 36, 54–57)
**MMP-1**	Leukocyte	Immune cell recruitment, collagen degradation	(58, 59)
**MMP-2**	Leukocyte	Immune cell recruitment, collagen degradation	(58, 60)
**MMP-3**	Leukocyte	Immune cell recruitment, connective tissue degradation	(58, 61)
**MMP-9**	Leukocyte	Immune cell recruitment (at high concentrations), inhibition of immune cell recruitment (at low concentrations)	(58)
**PDGF**	DC, FLS, macrophage	Immune cell recruitment, T_reg_ polarization, pannus formation, cartilage degradation, inhibition of DC maturation	(56, 62–65)
**TGF-β1 &** **TGF-β2**	FLS, macrophage, CD4^+^ T cell, B cell	Activation of immune cells, angiogenesis, cartilage degradation, osteoclastogenesis	(36, 62, 66–70)
**TSP-1**	Monocyte, macrophage, endothelial cell, neutrophil, DC, CD4^+^ T cell	Immune cell recruitment, T_reg_ formation, inhibition of antigen presentation, inhibition of angiogenesis, inhibition of neutrophil elastase	(71–80)
**VEGF**	Endothelial cell, monocyte, macrophage, FLS	Angiogenesis, immune cell recruitment and activation, osteoclastogenesis	(81–84)
**vWF**	Leukocyte	Immune cell recruitment	(85, 86)
**Glutamate**	T cell, FLS	Pro-inflammatory cytokine secretion, T cell activation (at low concentrations), inhibition of T cell activation (at high concentrations)	(87–89)
**Histamine**	FLS, endothelial cell	Endothelial activation, immune cell recruitment, FLS proliferation, MMP production	(90–92)
**NO**	FLS	Inhibition of FLS apoptosis	(93, 94)
**Serotonin**	CD4^+^ T cell, neutrophil	Immune cell recruitment and activation, ROS production	(95–97)
**TXA_2_**	Endothelial cell, FLS	Endothelium activation, FLS proliferation	(98–100)

ADP, adenosine diphosphate; CCL, chemokine C-C motif ligand; CD, cluster of differentiation; sCD62P, soluble P-selectin; CXCL, chemokine C-X-C motif ligand; CXCL4L1, non-allelic variant of CXCL4; DC, dendritic cell; FLS, fibroblast-like synoviocyte; IL, interleukin; MMP, matrix metalloproteinase; NO, nitric oxide; PAF, platelet activating factor; PDGF, platelet-derived growth factor; PGE_2_, prostaglandin E_2_; RA, rheumatoid arthritis; ROS, reactive oxygen species; sCD40L, soluble cluster of differentiation 40 ligand; TGF, transforming growth factor; T_reg_, regulatory T cell; TSP-1, thrombospondin-1; TXA_2_, thromboxane A_2_; VEGF, vascular endothelial growth factor; vWF, von Willebrand factor.

### A. Platelet activation and granule secretion

There are three types of platelet granules: alpha (α)-granules, dense granules, and lysosomes (reviewed in (102)). The first, and most abundant, are the α-granules that contain a multitude of cytokines, chemokines and growth factors (103). For example, α-granules release multiple pro-inflammatory molecules, including chemokine (C-C motif) ligand 5 (CCL5), soluble CD40 ligand (sCD40L/sCD154), and CXCL4 (aka platelet factor 4, PF4) (104). Furthermore, P-selectin (CD62P), a biomarker of α-granule secretion and platelet activation (105), is translocated from α-granules to the surface membrane upon platelet activation. Surface P-selectin promotes activation of leukocytes including neutrophils (see Platelet-neutrophil interactions section below). The second type of granule is the dense granule, so named due to its electron-dense appearance (102). The primary cargo of dense granules are nucleotides (adenosine diphosphate (ADP) and adenosine triphosphate (ATP)) and polyphosphates (102). Dense granule components primarily regulate platelet hemostasis and thrombosis (106). However, dense granules also release serotonin, which can promote inflammation by stimulating lymphocytes and neutrophils (95). The third type of granule is the lysosome, which contains tissue-degrading enzymes (such as collagenase) and acid phosphatase. As observed in other cell types, the key role of platelet lysosomes is to orchestrate catalytic breakdown of proteins (102).

### B. Platelet extracellular vesicles

In addition to their granular contents, activated platelets release two types of extracellular vesicles: exosomes and microparticles (or microvesicles) (107). Firstly, exosomes are 30-100 nm vesicles contained within, and released with, larger intracellular vesicles. Secondly, microvesicles or platelet microparticles (PMPs) have a diameter of 100-1000 nm and are formed by the outward blebbing of the plasma membrane. PMPs are derived from the platelet plasma membrane and therefore contain the same markers found on platelet surfaces, including CD42 (glycoprotein Ib) and CD41/61 (integrin αIIbβ3) (107). Importantly, PMPs levels are increased in RA and may drive RA progression *via* release of their pro-inflammatory contents, e.g., interleukin-1 (IL-1) (12, 107).

### C. Platelets are sentinels of the innate immune system

Platelet-derived cargo molecules (**Table 1**) include pro-inflammatory factors linked with chronic disorders including arthritis and inflammatory bowel disease (12, 108). Notably, platelets interact directly with bacteria (109), viruses (110) and complement proteins (111) and as such, are essential components of innate immunity. Platelets express toll-like receptors (TLRs) that sense pathogenic molecules (112) such as bacterial lipopolysaccharides (LPS) that induce platelet activation, aggregation, and leukocyte chemotaxis (113). Thus, platelets, as innate immune effectors, are recognized as critical mediators of RA pathogenesis (114, 115).

### D. Evidence of platelet involvement in RA

Early correlational evidence suggested a role for platelets in the development and progression of RA, in that platelets were reportedly recruited to joints with their concentration in synovial fluid correlating with disease activity (13, 116, 117). Later research indicated that elevated circulating platelet count (aka thrombocytosis) was associated with more severe RA (118). Subsequently, seminal research identified PMPs (12) in the synovial fluid of RA patients. Compared to patients with non-inflammatory arthritis, RA synovial fluid contains more IL-1-rich PMPs (12). Taken together, these data indicate that platelet numbers are increased in RA, and that cytokine-laden PMPs are heavily recruited to arthritic joints.

In addition to higher platelet counts, circulating levels of platelet agonists are elevated in RA patients. For example, fibrinogen levels correlate with the number of swollen joints and overall disease activity (119). Thromboxane A_2_ (TXA_2_) is another platelet agonist whose degradation product, TXB_2_, is correlated with disease activity in RA (98). These data are consistent with elevated platelet activity during active RA (120). This notion is further corroborated by evidence of elevated soluble P-selectin (121) and sCD40L (122) in RA plasma. Soluble CD40L levels correlate with autoantibody levels in RA patients (123). Collectively, these data raise the possibility that platelet-derived pro-inflammatory products and cytokines play a role in propagating RA-associated joint inflammation. Animal studies provide a degree of direct evidence for the involvement of platelets in the RA pathogenesis. For example, platelet depletion prior to the induction of arthritis in mice reduces disease severity (12). Therefore, understanding the precise mechanisms by which platelets drive synovial inflammation have important implications for prospective therapies.

### E. Molecules upregulated in RA promote platelet activation

In addition to driving RA pathogenesis, platelets can also be reciprocally activated by molecules that are elevated in RA plasma or joint tissue. For example, a central feature of RA is the generation of ACPA and formation of immune complexes nucleated around citrullinated proteins (11). Immune complexes bind and activate platelet Fc gamma receptor IIa (FcγRIIa) (124). Platelets are also activated by complement fragments (111). As such, complement activation during RA (125) can conceivably create a feedback loop through which platelet activity is continually elevated. Collectively, these data point to a two-way relationship between platelet function and innate responses in RA, where platelet-derived cytokines contribute to inflammation (**Figure 1B**) and ACPA-immune complexes (and other molecules) sustain platelet activation (**Figure 1A**). Moreover, RA-associated inflammatory mediators likely circulate and prime platelets systemically. These mechanisms may explain why RA patients are at higher risk for life-threatening complications such as deep vein thrombosis, myocardial infarction, and stroke (126, 127).

## Platelet-driven signaling in the synovial tissues

### A. Platelet growth factor signaling to FLS

Platelet α-granules store multiple forms of platelet-derived growth factor (PDGF) (128, 129), including the hetero and homo A/B dimers (PDGF-A/B) (130). PDGF-BB was recently confirmed to circulate in higher concentrations in RA patients (131). Furthermore, the invasiveness and proliferation of human FLS is increased following treatment with PDGF (62, 132). PDGF also promotes FLS production of pro-inflammatory cytokines IL-6, IL-8, as well as MMP-3 (133). In addition, platelets α-granules are an abundant source of transforming growth factor beta 1 (TGF-β1) (134). TGF-β1 stimulates the migratory behavior and invasiveness of cultured FLS obtained from RA patients (135). Interestingly, TGF-β reportedly acts synergistically along with PDGF to trigger FLS proliferation and a pro-inflammatory phenotype (132, 133). Collectively, these data provide both clinical and *in vitro* evidence of how synovial inflammation is likely propagated by platelet growth factors.

### B. Platelet-derived cytokines and FLS

In addition to growth factors, platelet granules release several pro-inflammatory cytokines that exacerbate RA disease activity (**Table 1**). For example, platelets release copious quantities of CCL5 (RANTES) (26). CCL5 drives RA pathogenesis by upregulating MMPs in RA FLS (136). Activated platelets also secrete IL-1β (12, 54). IL-1β promotes synovial inflammation and RA progression by upregulating RANKL expression by FLS (55), which, in turn drives osteoclastogenesis and bone resorption (137). PMPs are another source of IL-1α (12), IL-1β (12), CCL5 (138), and sCD40L (139). Moreover, the role of PMPs in RA is supported by the observation that they increase the invasiveness and motility of cultured FLS (140, 141). Taken together, the evidence supports a role for platelet-derived cytokines and PMPs in synovial tissue degradation by promoting the release of MMPs and additional pro-inflammatory cytokines from FLS.

## Interplay between platelets and other elements of the innate immune system

In addition to modulating disease by signaling to FLS, platelets interface directly with several other aspects of the innate immune system (**Figure 1B**). Platelets activate complement proteins and recruit leukocytes to the joint tissues. Multiple lines of evidence suggest that the pro-inflammatory leukocyte phenotype is enhanced *via* association with platelets. Here we detail the interactions between platelets and other aspects of the innate immune system that could explain the molecular basis underpinning the contribution of platelets to RA.

### A. Platelet-neutrophil interactions

#### Platelets promote neutrophil chemotaxis

A feature of RA pathogenesis is the marked influx of immune cells, including neutrophils, into the synovium (14). Evidence from human (142) and murine (143) studies indicate that neutrophils are important for RA development by participating in joint tissue degradation and by releasing proinflammatory cytokines that further exacerbate RA (144, 145). Platelets can potentially recruit neutrophils into the joint by four possible mechanisms. Firstly, platelet-derived serotonin (95) and CXCL7 (146) are direct neutrophil chemoattractants; platelets stimulate FLS to secrete IL-8, CXCL2, and CXCL3 (147), all of which attract neutrophils (148); PMPs also recruit neutrophils to the joints (149). Secondly, exposed P-selectin on the platelet surface facilitates neutrophil adhesion *via* P-selectin glycoprotein ligand 1 (PSGL1) on the neutrophil surface (150). Thirdly, platelet-derived membrane-bound platelet activating factor (PAF) activates the neutrophil PAF receptor, leading to the activation of CD18 on the neutrophil surface (151). Neutrophil CD11b/CD18 (aka Mac-1) can then bind to glycoprotein Ibα (GPIbα) on the platelets (152). Finally, both neutrophil-derived CD11b/CD18 (153) and platelet-derived integrin αIIbβ3 (154) bind fibrinogen; CD11b/CD18 and αIIbβ3 can therefore mediate platelet-neutrophil adhesion *via* fibrinogen. These data provide compelling evidence of platelet-neutrophil crosstalk that can drive RA pathogenesis.

#### Platelets promote NET formation

Another central feature of neutrophils in the innate immune response is the release of neutrophil extracellular traps (NETs) consisting of genomic DNA, proteolytic enzymes, and reactive oxygen species (ROS) (155). NETs contribute to self-protein citrullination and correlate with ACPA levels (142) (**Figure 1C**). Platelets colocalize with degranulated neutrophils and NETs in coronary thrombi (156). Moreover, platelet depletion restricts NET formation in mouse models of endotoxemia (157). Activated platelets releases cytosolic stores of the alarmin high mobility group box 1 protein (HMGB1) that activates receptor for advanced glycation end products (RAGE) expressed on the surface of neutrophils, causing neutrophil autophagy and subsequent NET formation (156). These evidences implicate platelets as pivotal supporters of neutrophil-mediated inflammation and tissue degradation in RA.

### B. Platelets interactions with the complement system

The complement system is an essential component of the innate immune response and is comprised of 9 serum proteins (C1-C9) activated sequentially, culminating in the formation of the membrane attack complex (MAC) that eliminates pathogens but is also implicated in autoimmune pathogenesis [reviewed in (158)]. There exist three separate pathways to complement activation: the classical pathway (elicited by binding of immunoglobulin to C1), the alternative pathway (triggered by the hydrolysis of C3 producing C3-H_2_O) and the lectin pathway (111).

Considerable evidence implicates complement activation as a contributing factor in RA [recently reviewed in (159)]. For example, MAC is upregulated in RA serum and synovial fluid (125), while an endogenous inhibitor of MAC, CD59, is downregulated in RA synovium (160). In addition, the role of complement proteins in neutrophil activation (161) is likely related to RA progression since neutrophils contribute to tissue degradation (144, 145) (**Figure 1C**).

Platelets contribute to complement activation in multiple ways. For example, a leukocyte-derived molecule termed properdin binds the platelet plasma membrane and recruits C3(H_2_O) thus activating the alternative pathway (162). Moreover, P-selectin on activated platelets binds C3b (163). Activated platelets also bind ficolin, which stimulates the lectin pathway (164). The evidence collectively supports a role for platelets in RA pathogenesis *via* the complement system.

### C. Platelet interactions with monocytes/macrophages

Macrophages phagocytose invading pathogens and as such are critical elements of the innate immune response. As reviewed by Ley (165), macrophages may be polarized to a proinflammatory (M1) or an anti-inflammatory (M2) phenotype; in addition, M1 macrophages exacerbate bone degradation in RA. Accordingly, the number of macrophages in the synovium correlates with RA severity in humans (166), and depletion of monocytes attenuates the development of experimental murine arthritis (167).

#### Platelets and monocyte chemotaxis

As is observed with neutrophils, platelets serve to recruit monocytes. First, monocytes adhere to endothelium-bound platelets through the interaction between platelet surface P-selectin and PSGL1 expressed on the monocyte surface (168). Second, platelet-derived molecules promote adhesion between monocytes and the endothelium. Platelet-derived CCL5, CXCL4 (platelet factor 4, PF4), and CXCL7 promote monocyte chemotaxis as well as monocyte-endothelium adhesion (37). These chemokines and the anaphylatoxin C5a (generated by platelet-induced complement activation) upregulate CD11b (169), β1 integrin (170), and CD18 (β2 integrin) (170) on the monocyte surface. CD11b/CD18, which is expressed by monocytes (168), binds to platelet-derived junction adhesion molecule C (JAMC/JAM3) (171) and GPIbα (172) to facilitate firm adhesion.

#### Platelet-monocyte interactions

Once inside the joint, platelet-monocyte interactions contribute to joint pathology. *In vitro* experiments show that PAF, P-selectin, and CCL5 (RANTES) induce the secretion of CCL2, TNF-α, and IL-8 from monocytes (27, 173); these mediators can then in turn upregulate inflammation in the joint. Moreover, CXCL4 (PF4), a major platelet-derived chemokine, polarizes macrophages to an M4 subtype (distinct from M1 and M2 subtypes) that secretes pro-inflammatory TNF-α (38) and generates reactive oxygen species (ROS) (39). In addition, the differentiation of monocytes into bone-resorbing osteoclasts is accentuated by platelet-derived TGF-β1 (66). Taken together, these data illuminate multiple pathways through which platelets could promote synovial inflammation through interactions with monocytes/macrophages.

### D. Platelet interactions with dendritic cells

Dendritic cells (DCs) are immune cells whose morphology is characterized by tree-like (dendritic) processes (174). Activated dendritic cells present pathogen-derived antigens to T cells, thus acting as a bridge between the innate and adaptive immune responses (175). Evidence suggests that DCs play an important role in RA (176). As is the case with neutrophils and monocytes, evidence supports a potential role for platelets in recruiting DCs. For example, *in vitro* data indicate that platelet-derived P-selectin and JAMC bind to PSGL1 and CD11b/CD18 expressed on DCs to promote platelet-DC adhesion (40). However, the platelets’ effects on DCs in disease are ambiguous since platelet-DC interactions can either promote or dampen inflammation. Platelet-derived sCD40L and CXCL4 (PF4) induce DC maturation, pro-inflammatory cytokine secretion from DCs, and interferon-α (IFN-α) secretion from stimulated plasmacytoid DCs (pDCs) *in vitro* (40, 177, 178). IFN-α causes DCs to secrete more TNF-α upon TLR4 stimulation, exacerbating RA (179). However, IFN-α can induce the polarization of both pro-inflammatory (180) and anti-inflammatory (181) T-cells, which can worsen or alleviate RA. Moreover, platelets also influence DC-mediated proliferation of T-cells (40) although it is not clear whether these T-cells adopt a pro-inflammatory (Th1/Th17) or anti-inflammatory regulatory (Treg) phenotype (**Figure 1C**). Further research is required to clarify the significance of DC cell function in RA pathogenesis and determine how DC function could be regulated through interactions with platelets.

### E. Platelet interactions with innate lymphoid cells

Innate lymphoid cells (ILCs) include five subsets of antigen-independent effector and helper cells that mirror the phenotypes of T-cell subsets (182). This group includes natural killer (NK) cells, lymphoid tissue inducer (LTi) cells and three subtypes of innate lymphoid ‘helper-like’ cells [ILC1, ILC2 and ILC3, reviewed in (183)]. ILCs have developmental, tissue-specific, and context-dependent roles in immunity with considerable phenotypic heterogeneity that are still under investigation (183, 184). Thus, the full picture of ILC biology, particularly in the context of RA [recently reviewed in (185)], is incomplete. Generally, the ILC subsets (NK/ILC1, LTi, ILC3 cells) that promote Th1/Th17-like responses, M1-macrophage polarization or recruitment of neutrophils likely exacerbate RA (185). Conversely, ILC2s, which promote Th2/Treg-like immunity, M2-polarization, and recruitment of eosinophils likely attenuate RA severity (185).

The role of platelets in modulating ILC function is poorly understood. ILC2 cell counts are elevated in the circulation and the synovial fluid of RA patients compared to healthy controls although their presence was inversely correlated with disease severity (186). In mouse models of arthritis, ILC2 appear to diminish inflammation and bone destruction (186). Limited data are available regarding platelet interactions with ILC2 cells. Naïve ILC2 cells express PSGL-1 and adhere to platelets in murine lung tissue, presumably *via* P-selectin on the platelet surface (187, 188). This interaction maintains elevated ILC2 numbers in naïve lung, enhances response to IL-33, amplifies production of Th2-cytokines (IL-5 and IL-13) and, exacerbates lung inflammation in response to *Alternaria* (fungus) (187, 188). While documented ILC2-platelet adhesion is limited to lung tissue thus far (187), platelet-ILC2 interactions in the synovium could also conceivably attenuate RA. It is tempting to speculate that platelet-derived mediators would recruit and/or activate most ILC subsets, however, considerable further research is required to identify relevant platelet-ILC interactions in the synovium.

## Perspectives

Considerable evidence now indicates that activated platelets participate in pro-inflammatory signaling in the synovial tissues, in part through activating other cellular elements of the innate immune system. However, pro-inflammatory molecules in RA also potentiate platelet activity, suggesting a reciprocal relationship between RA and platelet activation. Platelets are therefore well-positioned to occupy a central role in RA pathogenesis as it relates to the innate immune response. Indeed, the concept of platelets as a therapeutic target for RA has been explored (189) although due to the nature of many of the currently available antiplatelet drugs, it may be difficult to exploit the anti-inflammatory effects without conferring increased bleeding risks. Moreover, the sheer complexity of the biological system (**Figure 1B**) highlights the challenges associated with pinpointing the most clinically significant platelet-centric signaling pathways in RA. Since multiple pro-inflammatory cytokines are expressed in more than one tissue type, future research could employ experimental murine models with platelet-specific conditional knockouts of specific cytokines (and/or their cognate receptors). Such an approach could identify the exact contributions of specific platelet-derived molecules to RA pathogenesis. This information would then be applicable for identifying viable platelet-based therapeutic targets that could reduce disease severity, and mitigate the increased risk of cardiovascular complications resulting from RA.

## Author contributions

SJ wrote the first draft of the manuscript. JT, MH, KM and HK designed the concept and layout of the manuscript and/or wrote sections of the manuscript. All authors contributed to the article and approved the submitted version.

## Funding

This work was supported by a Canadian Institutes of Health Research (CIHR) Operating Grant (MY4-155400) and a Michael Smith Foundation for Health Research (MSFHR) Scholar Award (to HK).

## Acknowledgments

The authors thank Angela Tether for editorial assistance. JT and SJ acknowledge support from a Graduate Student Award Program from the UBC Centre for Blood Research. SJ also acknowledges support from the UBC International Tuition Award.

## Conflict of interest

The authors declare that the research was conducted in the absence of any commercial or financial relationships that could be construed as a potential conflict of interest.

## Publisher’s note

All claims expressed in this article are solely those of the authors and do not necessarily represent those of their affiliated organizations, or those of the publisher, the editors and the reviewers. Any product that may be evaluated in this article, or claim that may be made by its manufacturer, is not guaranteed or endorsed by the publisher.
